# Glnk Mediates Carbapenem Resistance Through the NtrB/NtrC-OprD Regulatory Pathway in *Pseudomonas aeruginosa*

**DOI:** 10.3390/pathogens15030289

**Published:** 2026-03-06

**Authors:** Xiaomeng Sun, Yiming Li, Xuetao Gong, Qitong Du, Yongxin Jin, Zhihui Cheng, Shouguang Jin, Weihui Wu

**Affiliations:** State Key Laboratory of Medicinal Chemical Biology, Key Laboratory of Molecular Microbiology and Technology of the Ministry of Education, Department of Microbiology, College of Life Sciences, Nankai University, Tianjin 300071, China; 1120220629@mail.nankai.edu.cn (X.S.); mitchell.li@foxmail.com (Y.L.); 1120220627@mail.nankai.edu.cn (X.G.); 1120250938@mail.nankai.edu.cn (Q.D.); yxjin@nankai.edu.cn (Y.J.); zhihuicheng@nankai.edu.cn (Z.C.); nksjin@nankai.edu.cn (S.J.)

**Keywords:** *Pseudomonas aeruginosa*, GlnK, NtrB/NtrC, *oprD*, carbapenem antibiotics

## Abstract

*Pseudomonas aeruginosa* is a major causative agent of nosocomial infections worldwide. Carbapenems are the first-line agents for combating severe *P. aeruginosa* infections. However, the increasing prevalence of carbapenem-resistant *P. aeruginosa* (CRPA) has developed as a critical threat to global healthcare systems. In this study, we demonstrated that a mutation in the core nitrogen metabolism regulatory gene *glnK* decreases carbapenem resistance in *P. aeruginosa*. OprD, the major porin for carbapenem uptake, is upregulated in the *glnK* mutant, resulting in decreased resistance. We further found that the NtrB/NtrC two-component regulatory system is upregulated in the *glnK* mutant. An electrophoretic mobility shift assay (EMSA) and genetic studies revealed a direct regulatory role of NtrC on the expression of *oprD*. Deletion of *ntrB*, *ntrC*, or *oprD* in the *glnK* mutant restored the bacterial resistance to carbapenems. These results reveal that a GlnK-NtrB/NtrC-OprD regulatory pathway affects carbapenem resistance, shedding light on the regulatory relationship between nitrogen metabolism and carbapenem resistance in *P. aeruginosa.*

## 1. Introduction

Antimicrobial resistance (AMR) poses a tremendous threat to global public health. It compromises the clinical efficacy of antibiotics in combating bacterial infections, imposing overwhelming burdens on healthcare systems and elevating mortality rates of infectious diseases [1,2]. A global burden study revealed that 4.71 million deaths were associated with bacterial AMR between 1990 and 2021, and 1.14 million deaths were attributable to bacterial AMR in 2021. It was predicted that these figures would surge to 8.22 million AMR-associated deaths globally by 2050 [3]. AMR-related environmental risks are increasing sharply. The inappropriate use and overprescription of antibiotics in clinical and agricultural settings have exacerbated global AMR prevalence [4,5]. Among the multidrug-resistant pathogens driving this crisis, *Pseudomonas aeruginosa* stands out as one of the most problematic pathogens [6].

*P. aeruginosa* is an opportunistic pathogen notorious for its robust antibiotic resistance and ability to colonize and persist in diverse microenvironments, such as nosocomial settings and the lungs of cystic fibrosis patients [7]. It has a relatively large genome, which arms the bacterium with a repertoire of antibiotic resistance determinants [8,9]. These determinants enable the pathogen to evade the inhibitory or killing effects of antibiotics by preventing the entry or actively extruding antibiotics, enzymatically degrading or modifying antibiotics, and modifying cellular targets [10,11,12]. Additionally, spontaneous chromosomal mutations and the acquisition of genes conferring antibiotic resistance via horizontal transfer (HGT) further augment its resistance [13,14,15].

β-lactams are one of the most widely used classes of antimicrobial agents in clinical practice against bacterial infections. Characterized by a conserved core β-lactam ring, these antibiotics exert their activities by targeting penicillin-binding proteins (PBPs), the enzymes catalyzing peptidoglycan cross-linking [16]. Major subgroups of β-lactam antibiotics include penems, penicillins, carbapenems, cephalosporins, and monobactams, among which carbapenems are considered reliable antibiotics for severe *P. aeruginosa* infections [17]. However, *P. aeruginosa* has progressively developed resistance to carbapenems, primarily via mechanisms including overexpression of efflux pumps such as MexAB-OprM, mutations in their PBP genes, reduction of outer membrane permeability, and acquisition of carbapenemases [18,19,20,21,22]. The outer membrane protein OprD is a substrate-specific porin for the uptake of basic amino acids. It is also a major porin for the entry of carbapenems. Mutation of *oprD* has been found to lead to carbapenem-resistance. Among 382 carbapenem-resistant *P. aeruginosa* (CRPA) isolates collected from 78 hospitals across Japan from 2019 to 2020, 87.1% (333/382) strains harbored mutations in *oprD* [23]. Another study identified the *oprD* mutation in 97.1% (68/70) CRPA strains [24].

GlnK, a homologue of the PII signal protein GlnB, functions as a core regulator of nitrogen metabolism in *P. aeruginosa*. Unlike many other bacteria that encode both GlnK and GlnB PII signal proteins, *P. aeruginosa* only harbors GlnK. In response to nitrogen fluctuations, the GlnK protein undergoes post-translational modification, altering its interaction with its cognate binding partners and thus modulating key metabolic pathways [25]. For instance, GlnK interacts with the ammonia transport protein AmtB to fine-tune ammonia transport [26]. Studies in *E. coli* demonstrate that GlnK regulates the activity of NtrB, the master regulator of nitrogen metabolism, to exert hierarchical control over nitrogen metabolic processes [27]. NtrB functions as both a kinase and a phosphatase, catalyzing the phosphorylation and dephosphorylation of NtrC. In *E.coli*, this dual enzymatic activity is governed by GlnK in a post-translational modification-dependent manner, in which unmodified GlnK interacts with NtrB, inhibiting its kinase activity and activating the phosphatase activity. In contrast, modified GlnK is unable to interact with NtrB, thereby permitting NtrB to phosphorylate NtrC [28]. Phosphorylated NtrC activates the nitrogen (*ntr*) regulon, such as the glutamine synthetase gene *glnA*, and coordinates with CbrB to regulate histidine utilization (*hut*) genes [27,29]. These regulatory mechanisms enable bacteria to dynamically optimize carbon-nitrogen metabolism in response to diverse nutrient conditions, which is critical for their colonization in the host.

It has been reported that nutritional conditions trigger global metabolic responses in bacteria, which affect antibiotic resistance [30]. Carbon sources have been shown to tune tobramycin tolerance in *P. aeruginosa* [31]. In addition, distinct nutrient cues shape the evolution of resistance of *P. aeruginosa* [32,33]. These results demonstrate a functional link between nutrient metabolism and antibiotic resistance. However, the role of carbon-nitrogen metabolic balance in bacterial antibiotic resistance remains largely unknown.

Here, we demonstrate that GlnK modulates *P. aeruginosa*’s carbapenem susceptibility by regulating the NtrB*/N*trC two-component system and subsequent OprD. Our findings reveal a regulatory link between metabolism and carbapenem resistance.

## 2. Materials and Methods

### 2.1. Plasmids, Primers, and Bacterial Strains

The plasmids, primers, and bacterial strains used in this study are summarized in Table 1. Bacteria were grown aerobically in LB broth (5 g/L yeast extract, 10 g/L tryptone, and 5 g/L NaCl, pH 7.4) at 37 °C and 200 rpm. When needed, antibiotics were added at the following concentrations: for *E. coli*, 10 μg/mL tetracycline, 100 μg/mL ampicillin, and 10 μg/mL gentamicin; for *P. aeruginosa*, 50 μg/mL tetracycline, 150 μg/mL carbenicillin, and 50 μg/mL gentamicin.

### 2.2. Construction of the glnK Mutant

The *glnK* mutant was constructed as described earlier, with slight modifications [38]. Briefly, the *glnK* deletion mutant was constructed using the suicide vector pEX18Tc via homologous recombination. Approximately 1 kb DNA fragments upstream and downstream of the *glnK* gene were amplified from wild-type *P. aeruginosa* PA14 genomic DNA using primer pairs *glnK*-LF/*glnK*-LR and *glnK*-RF/*glnK*-RR, respectively, and fused by overlap PCR. The resulting PCR product fragment was digested with Sac I and BamH I, and cloned into the pEX18Tc. The resulting recombinant plasmid was transformed into *E.coli* S17-1 by electroporation and then mobilized into *P. aeruginosa* PA14 by conjugation. Transconjugants were selected on LB agar containing tetracycline (50 μg/mL, to counterselect wild-type PA14) and kanamycin (25 μg/mL, to counterselect *E. coli* S17-1). These single-crossover integrants were then streaked onto LB agar supplemented with 7.5% sucrose. Sucrose-resistant colonies were screened for the *glnK* deletion mutant using colony PCR with primers *glnK*-LF and *glnK*-RR. Other deletion mutants were constructed using the same strategy.

### 2.3. Minimum Inhibitory Concentration (MIC) Assay

For the MIC determination assay, the standard broth microdilution approach was carried out in triplicate, in accordance with the Clinical and Laboratory Standards Institute (CLSI) guidelines. Briefly, antibiotics were two-fold serially diluted in cation-adjusted Mueller–Hinton broth (CAMHB, 3.0 g/L beef extract, 17.5 g/L casamino acid, 1.5 g/L soluble starch, 0.5 mM CaCl_2_, 0.5 mM MgCl_2_, and pH 7.3 ± 0.1), followed by mixing with an equal volume of bacterial suspension in a 96-well microplate. The final bacterial concentration was adjusted to 5  ×  10^5^ CFU/mL. MIC values were defined as the lowest concentration of antibiotics yielding no visible bacterial growth after incubation at 37 °C for 20 h. The assays were performed with three biological repeats. Since the antibiotics were two-fold serially diluted, the standard deviations were calculated with log_2_-transformed MICs (log_2_MIC).

### 2.4. Ethidium Bromide Influx Assay

The assay was performed as previously described with slight modifications [39,40]. Bacteria were cultured in LB medium until OD_600_ reached 0.7, and then washed and resuspended in PBS supplemented with 0.5% (*m*/*v*) glucose. The cells were incubated with 100 μM of carbonyl cyanide m-chlorophenylhydrazone (CCCP) or not, and 1 μg/mL of ethidium bromide, followed by incubation for 30 min at 37 °C. The accumulation of ethidium bromide was measured using Varioskan™ Flash (ThermoFisher, Waltham, MA, USA) with an excitation wavelength of 530 nm and an emission wavelength of 585 nm. The relative uptake of ethidium bromide was normalized to the OD_600_ of each sample.

### 2.5. RNA Extraction and Real-Time Quantitative PCR

Overnight cultured cells were diluted 1:50 in LB and incubated at 37 °C to an OD_600_ of 1.0. Total RNA was isolated using the TransZol Up Plus RNA Kit (TransGen Biotech, Beijing, China) according to the manufacturer’s instructions. Complementary DNA (cDNA) was then synthesized from respective RNA samples utilizing HiScript III RT SuperMix for qPCR (Vazyme, Nanjing, China), followed by amplification with Super Multiple Probe qPCR PreMix (Vazyme, Nanjing, China). The 30S ribosomal protein gene *rpsL*, a well-validated housekeeping gene for internal controls in *P. aeruginosa*, was utilized for normalization of gene expression levels [41].

### 2.6. β-Galactosidase Activity Assay

The β-galactosidase activity assay was conducted according to a previous protocol, with slight modifications [42]. Bacteria were cultured in LB at 37 °C with shaking at 200 rpm until the OD_600_ reached 1.0, and then cells were harvested and resuspended in an equal volume of LacZ-buffer (0.04 M NaH_2_PO_4_, 0.06 M Na_2_HPO_4_, 0.001 M MgSO_4_, 0.01 M KCl, and 0.05 M β-ME). A 500 μL aliquot of the suspension was mixed with 10 μL of 0.1% (*m*/*v*) SDS and 10 μL of chloroform, followed by vortexing for 10 s to lyse the cells. Subsequently, orthonitrophenyl—galactopyranoside (ONPG, 4 mg/mL, BBI Life Sciences, Shanghai, China), at a volume of 100 μL, was added and incubated at 37 °C. The reaction was stopped by adding 500 μL of 1 M Na_2_CO_3_ solution once the reaction mixture turned yellow. The calculation of β-galactosidase activity was as previously described [43].

### 2.7. Electrophoretic Mobility Shift Assay (EMSA)

The electrophoretic mobility shift assay (EMSA) was performed with slight procedural modifications based on a previous protocol [44]. Briefly, DNA fragments amplified by PCR from the *P. aeruginosa* PA14 genome were incubated with purified recombinant NtrC protein in a 20 μL reaction buffer (100 mM Tris–HCl, pH 7.4, 5 mM EDTA, 500 mM KCl, 35 mM MgCl_2_, and 5 mM DTT) for 30 min at 25 °C. Electrophoresis was carried out at 10 mA for 60 min in 0.5 × TBE buffer after loading the samples onto an 8% native polyacrylamide gel. The entire process was carried out on ice.

### 2.8. Statistical Analysis

All analyses were performed using GraphPad Prism 8.0 (San Diego, CA, USA), and data are presented as the mean ± SD. Comparisons between groups were conducted using Student’s *t* test or one-way ANOVA followed by Dunnett’s multiple comparison test. A *p*-value less than 0.05 was considered statistically significant.

## 3. Results

### 3.1. Susceptibility of the glnK Mutant to Carbapenem Antibiotics

To investigate the role of *glnK* in β-lactam resistance, we measured the MICs of various subclasses of β-lactam antibiotics, including carbapenems, cephalosporins, monobactam, penicillin, and cephamycin for wild-type PA14, the *glnK* mutant (Δ*glnK),* and the complemented strain (Δ*glnK*/att7::*glnK*). The Δ*glnK* mutant exhibited a two- to four-fold increased susceptibility to carbapenem antibiotics, including meropenem (MEM), imipenem (IMP), and doripenem (DRPM), compared with the wild-type strain PA14, and this susceptibility was restored by complementation with the *glnK* gene (Δ*glnK*/att7::*glnK*) (Table 2). In contrast, no significant changes in MICs were observed for other β-lactam subclasses. These results indicate a role of GlnK in carbapenem resistance.

### 3.2. Upregulation of OprD in the glnK Mutant Contributes to the Increased Carbapenem Susceptibility

**Figure 1:**
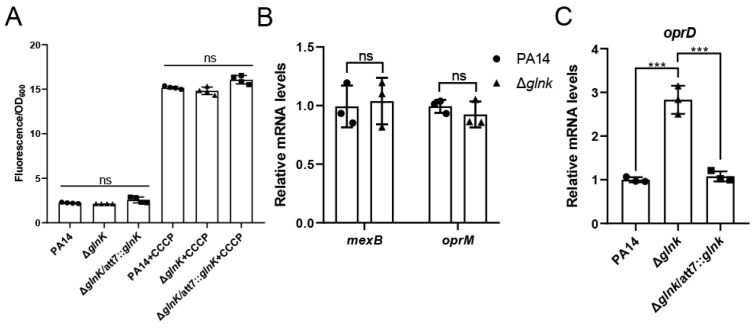
Upregulation of *oprD* in the Δ*glnK* mutant contributes to the susceptibility to carbapenem antibiotics. (**A**) Ethidium bromide (EB) accumulation in the indicated strains. A total of 100 μM carbonyl cyanide m-chlorophenylhydrazone (CCCP) was used as the control. (**B**) mRNA levels of *mexB* and *oprM* in the wild-type PA14 and Δ*glnK*. Gene expression levels were normalized to *rpsL* mRNA levels. (**C**) mRNA levels of *oprD* in the indicated strains. *oprD* mRNA levels were normalized to *rpsL* mRNA levels. Data are shown as mean ± SD from triplicate or quadruplicate biological replicates. ***, *p* < 0.001; ns, not significant by one-way ANOVA.

To explore the mechanism of *glnK*-mediated carbapenem resistance, we first measured the bacterial efflux activity by measuring intracellular accumulation of ethidium bromide (EB) [45]. The EB influx assay is a well-established method for the functional assessment of efflux pumps [46,47], in which reduced efflux activity results in increased intracellular EB accumulation, thereby enabling reliable comparison of efflux pump function among isogenic strains. In this assay, PA14, Δ*glnK*, and Δ*glnK*/att7::*glnK* treated with carbonyl cyanide m-chlorophenylhydrazine (CCCP), an efflux pump inhibitor, served as the control. In the absence of CCCP, intracellular EB accumulation was comparable among the strains of PA14, Δ*glnK*, and Δ*glnK*/att7::*glnK*, while CCCP addition elevated the intracellular EB levels in all strains to a similar level (Figure 1A), indicating that efflux pump activity was comparable among these strains. Given that MexAB-OprM is the major efflux system for the extrusion of carbapenems from the cell interior [48]. We further examined the gene expression levels of *mexB* and *oprM* by RT-qPCR. Consistent with the efflux activity result, no significant difference in *mexB* and *oprM* expression was observed between PA14 and the Δ*glnK* mutant (Figure 1B). In addition, the expression levels of other efflux pump genes (*mexC*, *mexE,* and *mexY*) were similar between PA14 and the Δ*glnK* mutant (Supplementary Figure S1). Together, these results suggest that the efflux activity is not responsible for the increased carbapenem susceptibility of the Δ*glnK* mutant. In *P. aeruginosa*, the basic amino acid porin OprD is a major channel for the diffusion of the carbapenem antibiotics [49,50]. Differential gene expression (DGE) analysis revealed that the *oprD* gene was upregulated in the Δ*glnK* mutant (Supplementary Table S1B). RT-qPCR results confirmed the upregulation of *oprD* in the Δ*glnK* mutant. Complementation with the *glnK* gene restored the expression level of *oprD* (Figure 1C). To examine the role of OprD in the increased carbapenem susceptibility of the Δ*glnK* mutant, we deleted the *oprD* gene. In both wild-type PA14 and Δ*glnK* mutant, deletion of *oprD* increased the MICs of the carbapenem antibiotics by 8- to 256-fold, resulting in the same MICs in the Δ*oprD* and Δ*glnKoprD* mutants (Table 3). These results demonstrate that the upregulated *oprD* contributes to the increased carbapenem susceptibility in the Δ*glnK* mutant.

### 3.3. GlnK Regulates oprD Through the NtrB/NtrC Two-Component System

**Figure 2:**
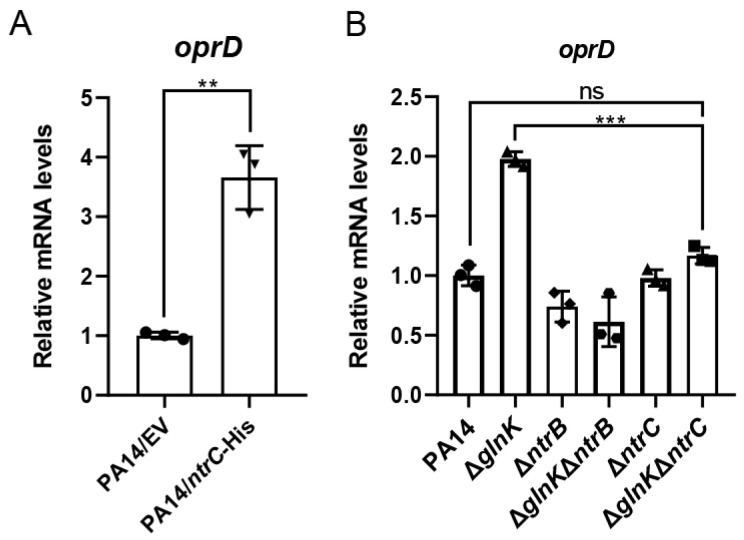
GlnK regulates *oprD* via the NtrB/NtrC two-component regulatory system. (**A**) mRNA levels of the *oprD* gene in PA14 and *ntrC*-overexpressing PA14. *oprD* mRNA levels were normalized to *rpsL* mRNA levels. EV, empty vector. **, *p* < 0.01 by Student’s *t*-test. Data are from three biological repeats. (**B**) mRNA levels of the *oprD* gene in the indicated strains. *oprD* mRNA levels were normalized to *rpsL* mRNA levels. Data are shown as means ± SD from triplicate biological replicates. ***, *p* < 0.001; ns, not significant by one-way ANOVA.

We previously found that the mutation of *glnK* resulted in upregulation of the *ntrB*/*ntrC* genes [35]. Since NtrB/NtrC regulates nitrogen metabolism genes, we hypothesized that GlnK might modulate *oprD* expression through this two-component regulatory system. To verify this hypothesis, we overexpressed the regulatory gene *ntrC* in PA14, which increased the expression of *oprD* (Figure 2A). Deletion of *ntrB* or *ntrC* in the Δ*glnK* mutant reduced the expression of *oprD* to a similar level as that in the wild-type PA14 (Figure 2). Furthermore, deletion of *ntrB* or *ntrC* in the Δ*glnK* mutant increased bacterial resistance to the carbapenem antibiotics (Table 4). Further deletion of *oprD* in the Δ*glnK*Δ*ntrB* mutant (Δ*glnK*Δ*ntrB*Δ*oprD*) resulted in a MIC comparable to that of the Δ*oprD* single mutant (Supplementary Table S2), demonstrating a major role of OprD in carbapenem resistance. Collectively, these results demonstrate that GlnK regulates *oprD* by modulating the NtrB/NtrC two-component system.

### 3.4. NtrC Directly Regulates oprD

**Figure 3:**
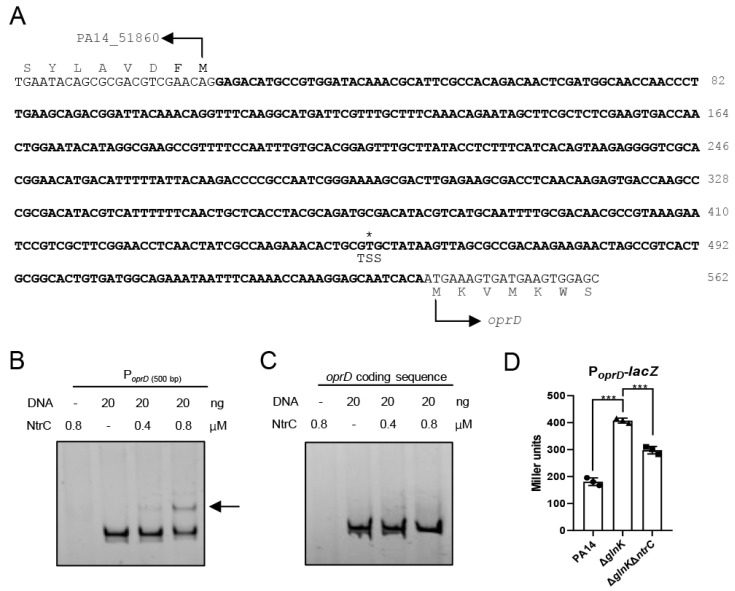
NtrC binds to the *oprD* promoter region. (**A**) Nucleotide sequences of the region between *oprD* and its upstream gene PA14_51860. The reported transcriptional start site of *oprD* is indicated by an asterisk above the nucleotide T and labeled TSS [51]. The promoter region cloned into the P*_oprD_*-*lacZ* is indicated in bold. TSS, transcription start site. (**B**,**C**) EMSA with purified NtrC and indicated probes. The shifted band is indicated by an arrow. (**D**) Promoter activities of *oprD*, PA14, Δ*glnK,* and Δ*glnK*Δ*ntrC* containing the P*_oprD_*-*lacZ* transcriptional fusion were cultured in LB and subjected to the β-Galactosidase activity assay. Data are shown as means ± SD from triplicate biological replicates and are representative of three independent experiments with similar results. ***, *p* < 0.001 by one-way ANOVA.

To determine whether NtrC directly activates the transcription of *oprD*, we performed an electrophoretic mobility shift assay (EMSA) using purified NtrC and a DNA fragment (500 bp) covering the *oprD* promoter region (Figure 3A). The DNA fragment was shifted by NtrC, whereas the fragment (500 bp) from the *oprD* encoding region was not shifted, indicating direct binding of NtrC to the *oprD* promoter region. Subsequently, we conducted an *oprD* promoter-*lacZ* transcriptional fusion. Compared to the wild-type PA14, LacZ expression was higher in the Δ*glnK* mutant, which was reduced by the deletion of the *ntrC* gene (Figure 3D). These findings indicate that NtrC directly regulates *oprD* in *P. aeruginosa.*

## 4. Discussion

In this study, we demonstrate that *P. aeruginosa* GlnK modulates the expression of *oprD* via the NtrB-NtrC two-component system, which affects bacterial resistance to carbapenem.

In proteobacteria, GlnK functions as a central integrator of nitrogen and energy signals that fine-tunes target-specific interactions to coordinate nitrogen metabolism. GlnK is post-translationally modified by the bifunctional uridylyltransferase/uridylyl-removing enzyme GlnD and interacts with various target proteins in response to cellular nitrogen status. GlnD catalyzes the uridylylation and deuridylylation of GlnK under nitrogen-limiting and replete conditions, respectively [52]. This reversible modification at the conserved tyrosine residue in GlnK’s T-loop dynamically changes its conformation and binding affinity for downstream targets [53]. Additionally, GlnK also senses cellular nitrogen and energy status by integrating with 2-oxoglutarate (2-OG), ATP, and ADP, which modulates its conformational state and subsequent interactions with target proteins [54]. In *E. coli*, GlnK interacts with ribonucleotide monophosphatase UmpH to inhibit its phosphatase activity toward uridine 5′-monophosphate [55]. The GlnK-UmpH complex is modulated by the GlnK uridylylation status, as well as the levels of its allosteric effectors ATP, ADP and 2-OG, with the interaction detectable in the presence of either ADP or ATP alone but abrogated when ATP is combined with 2-OG. Notably, uridylylated GlnK (GlnK∼UMP_3_) fails to interact with UmpH regardless of the presence of these molecules.

The two-component regulatory system (TCS) NtrB/NtrC is recognized as a core regulatory hub governing gene expression involved in nitrogen assimilation and utilization. Beyond its canonical function in nitrogen metabolism, increasing evidence has revealed the pleiotropic regulatory targets of NtrB/NtrC in *P. aeruginosa*, including factors involved in pathogenicity and antibiotic resistance. Alford et al. reported that double deletion of *ntrB* and *ntrC* led to a significant (~50%) reduction in abscess size relative to the wild-type LESB58 strain in a chronic abscess infection model [56]. The Δ*ntrBC* mutant exhibits impaired swarming motility and biofilm formation, which are associated with chronic infections [56]. Furthermore, RNA-seq results revealed elevated expression of T3SS genes in Δ*ntrB* and Δ*ntrC* mutants [56]. This result is consistent with our previous finding that mutation of *glnK* results in upregulation of *ntrB*/*ntrC*, thereby repressing T3SS gene expression [35]. These results support a role of NtrB/NtrC in regulating the acute and chronic infection lifestyles in *P. aeruginosa* [57]. In our study, genes involved in nitrite assimilation, ammonium uptake, glutamine and glutamate synthesis, and urea detoxification and assimilation were upregulated in the Δ*glnK* mutant (Supplementary Table S1A). Alford et al. demonstrate that deletion of *ntrB* or *ntrC* reduced the expression of these genes [56]. For instance, the nitrite reductase small subunit gene *nirD* was downregulated in the *ntrB* and *ntrC* mutants, while upregulated in the Δ*glnK* strain. Since we observed that the mutation of *glnK* results in upregulation of *ntrB* and *ntrC* [35], these results indicate that the upregulation of the genes in the Δ*glnK* mutant might be mediated through the NtrB/NtrC two-component regulatory system.

Besides virulence factors, NtrB/NtrC has also been found to influence antibiotic resistance determinants. The previous RNA-seq analysis demonstrated the upregulation of *muxABC* and *opmB* in the *ntrB* and *ntrC* mutant strains. The MuxABC-OpmB is a resistance-nodulation-cell division (RND)-type multidrug efflux pump system, which contributes to the bacterial resistance to aztreonam, macrolides, novobiocin, and tetracycline [58]. In addition, NtrB/NtrC has been reported to directly affect ciprofloxacin resistance [59]. The MIC of the fluoroquinolone ciprofloxacin was reduced by 8-fold in Δ*ntrB* and Δ*ntrC* strains and by ≥16-fold in the Δ*ntrBC* double mutant, whereas no significant differences in the MICs were observed for tobramycin, chloramphenicol, and tetracycline [59]. In this study, our findings revealed that the upregulation of *ntrB*/*ntrC* in the Δ*glnK* mutant increases bacterial susceptibility to carbapenems (Table 2 and Table 4), which is due to upregulation of OprD (Figure 1C and Figure 2).

OprD is a well-characterized outer-membrane porin in *P. aeruginosa*, which serves as a channel for basic amino acid transportation and plays a critical role in the entry of carbapenems. Mutations in the *oprD* gene confer carbapenem resistance, especially to imipenem [60]. One study performed genomic sequencing analyses on carbapenem-resistant *P. aeruginosa* (CRPA) strains, identified a high prevalence of *oprD* mutations with no significant variations in the expression of common carbapenemases or efflux pump activity, and demonstrated that the loss of OprD is sufficient for carbapenem resistance [61]. Consistent with these results, our results revealed that deletion of *ntrB* in the Δ*glnK* restored carbapenem resistance (Table 4). In clinical isolates, *oprD* missense or nonsense mutations are frequently identified. These mutations result in conformational changes in this porin, thereby conferring carbapenem resistance [49,62].

The expression of *oprD* is upregulated when arginine, histidine, glutamate, or alanine is used as the sole carbon source in a minimal medium supplemented with ammonium sulfate as the nitrogen source. Similarly, *oprD* is upregulated when glutamate or alanine is used as the nitrogen source [51]. Arginine-mediated *oprD* upregulation is attributed to the activation of the arginine-responsive repressor ArgR, which functions as a direct activator of the *oprD* gene [51]. Here, we identified the role of NtrB/NtrC in regulating the expression of *oprD.* Given the role of NtrB/NtrC in regulating nitrogen metabolism, it is likely that *P. aeruginosa* may exhibit different levels of carbapenem susceptibility under various nutritional conditions.

These findings highlight the critical relevance of the nitrogen metabolism regulator to bacterial carbapenem susceptibility, as specifically evidenced by an approximately four-fold reduction in the MIC of meropenem in the *glnK* mutant (Table 2). As a common empirical antibiotic, meropenem resistance is associated with increased treatment failure and mortality in cystic fibrosis (CF) patients with *P. aeruginosa* infections [63,64]. Of note, elevated amino acid levels are present in the airways of CF patients [65], which may modulate bacterial susceptibility to meropenem. In fact, it has been reported that sequential *P. aeruginosa* isolates collected over 3 years from a CF patient exhibited a striking shift in meropenem MIC, from 0.25 μg/mL to 4 μg/mL and finally to 64 μg/mL, which was accompanied by a significant deterioration in the patient’s pulmonary function [66]. Mechanistically, this altered carbapenem susceptibility was mainly due to the progressive downregulation of the outer membrane porin OprD. Thus, it is likely that under the CF patients’ lung environment, mutation of *oprD* does not affect bacterial growth, presumably due to the high amino acid levels.

## 5. Conclusions

This study identifies a regulatory pathway by which GlnK mediates carbapenem susceptibility in *P. aeruginosa*. We demonstrate that mutation of *glnK* leads to upregulation of *ntrB*/*ntrC*, which activates *oprD* expression, enhancing bacterial susceptibility to carbapenems. These findings establish a functional link between nitrogen metabolism and antibiotic resistance, expanding the understanding of the complex regulatory network governing *oprD* expression. Collectively, this GlnK-mediated regulatory pathway provides a clue for the development of strategies to enhance the efficacy of carbapenems for combating *P. aeruginosa* infections.

## Figures and Tables

**Table 1:** Bacterial strains, plasmids, and primers used in this study.

Strain/Plasmids/Primers	Description	Source/Purpose
PA14	Wild-type strain	[34]
Δ*glnK*	PA14 *glnK* gene deletion mutant	[35]
Δ*glnK*/att7::*glnK*	Δ*glnK* complementation with *glnK* inserted on chromosome, Tc^r^	[35]
Δ*oprD*	PA14 *oprD* gene deletion mutant	[36]
Δ*glnK*Δ*oprD*	PA14 *glnK* and *oprD* genes double deletion mutant	This study
Δ*ntrB*	PA14 *ntrB* gene deletion mutant	[35]
Δ*ntrC*	PA14 *ntrC* gene deletion mutant	[35]
Δ*glnK*Δ*ntrB*	PA14 *glnK* and *ntrB* genes double deletion mutant	[35]
Δ*glnK*Δ*ntrC*	PA14 *glnK* and *ntrC* genes double deletion mutant	[35]
Δ*glnK*Δ*ntrB*Δ*oprD*	PA14 *glnK, ntrB,* and *oprD* genes triple deletion mutant	This study
Plasmids		
pEx18Tc-Δ*glnK*	*glnK* gene deletion suicide plasmid; Tc^r^	[35]
pEx18Tc-Δ*ntrB*	*ntrB* gene deletion suicide plasmid; Tc^r^	[35]
pEx18Tc-Δ*ntrC*	*ntrC* gene deletion suicide plasmid; Tc^r^	[35]
pEx18Tc-Δ*oprD*	*oprD* gene deletion suicide plasmid; Tc^r^	[36]
pUC18T-mini-Tn7-Tc-*glnK*-his	chromosomal integration of *glnK*-his	[35]
pTNS3	helper plasmid encoding the TnsABCD transposase subunits; Ap^r^	[37]
pUCP20-P*_oprD_*-*lacZ*	lacZ with *oprD* promoter (500 bp); Ap^r^	This study
pMMB67EH-*ntrC*-his	expression driven by an inducible tac promoter; Gm^r^	[35]
Primers		
*glnK*-LF	CGAGCTCGAGTGCGATGGCCAGGT	*glnK* deletion
*glnK*-LR	TCGGTTGGGCGAAACTCTCTCCCGTGTT	*glnK* deletion
*glnK*-RF	GAGAGAGTTTCGCCCAACCGAACCCCCAAA	*glnK* deletion
*glnK*-RR	CGGGATCCCCATCGGACCGGCGGTG	*glnK* deletion
mini-*glnK*-F	CGAGCTCGCTGACGCAGGGGGCTTC	GlnK cloning
mini-*glnK*-R	CGGGATCCTTAGTGATGGTGATGGTGATGGATCGCGTCGGTATCGGTTTC	GlnK cloning
*ntrB*-LF *ntrB*-LR *ntrB*-RF *ntrB*-RR *ntrC*-LF *ntrC*-LR *ntrC*-RF *ntrC*-RR NtrC-F NtrC-R P*_oprD_*-F P*_oprD_*-R	CGAGCTCAGTTGCAACTGGTGCTGGACGGCAAACCCTACGGCACGCCC CTCTGATCGGCTCATGGGGCGGGCAGCTGTTCCAAGGTTGGGCAGG GGAACAGCTGCCCGCCCCATGAGCCGATCAGAGACCGTCTGGATCGTC CCAAGCTTGCTCCTGGGCGGCGCGGCTGAGGAAGTGCCGGGCCAG CGGGATCCTGGAGTACATGAACCCGGCAG GTGGATCAACGGGTCAATGCACTCCTTGTTCCAGGGGCA GTGCATTGACCGAATACCTGCCCAAGCCGTTCGAC CCAAGCTTCGAGCTGGTGATGAATGCCTCTGGAG CGGAATTCATGAGCCGATCAGAGACCGTCTG CGGGATCCTTAGTGATGGTGATGGTGATGGTCGTCGCCTTCGTCGTC CGGAATTCCAAACGCATTCGCCACAGACAAC CGGGATCCTGTGATTGCTCCTTTGGTTTTG	*ntrB* deletion *ntrB* deletion *ntrB* deletion *ntrB* deletion *ntrC* deletion *ntrC* deletion *ntrC* deletion *ntrC* deletion NtrC cloning NtrC cloning P*_oprD_*-lacZ cloning P*_oprD_*-lacZ cloning
Probe-*oprD*-F	CAAACGCATTCGCCACAG	EMSA
Probe-*oprD*-R Negative-*oprD*-F Negative-*oprD*-R	TGTGATTGCTCCTTTGGTTTTG GTGGAGCGCCATTGCACT CTTCGAGTTCGCTGCTCTG	EMSA EMSA EMSA
RT*-mexB-*F	GTGGGTGATCGCCTTGGTG	RT-PCR
RT*-mexB-R*	GCTCACCTGCACGGCGATG	RT-PCR
RT*-oprM-*F	CCGCCTACCTGACGCTGA	RT-PCR
RT*-oprM*-R	ACGCCGACGTCGTAGCTG	RT-PCR
RT*-oprD-*F	CACTCAGTTCGCCGTGGC	RT-PCR
RT*-oprD*-R	GCCGCTCTTGCCGTCACG	RT-PCR
RT-*mexE*-F	CACTTCTCCTGGCGCTAC	RT-PCR
RT-*mexE*-R	CTTCGGCGACGCTGACCT	RT-PCR
RT-*mexC*-F	GTTGGCAGGTTGTGGGCC	RT-PCR
RT-*mexC*-R	ACTCAGCGCCAGGGACTCG	RT-PCR
RT-*mexY*-F	CTGGCGATCCGCTTCCTGC	RT-PCR
RT-*mexY*-R	CCTCGACCACCTTGGCCGAGGC	RT-PCR

The restriction enzyme cleavage sites within the primers are underlined. Tc^r^, tetracycline resistance; Ap^r^, ampicillin resistance; Gm^r^, gentamicin resistance.

**Table 2:** Minimum inhibitory concentrations (MICs) of β-lactam antibiotics.

Strain	MIC ^a^ (μg/mL)							
	MEM ^b^	IMP ^c^	DRPM ^d^	FEP ^e^	CAZ ^f^	ATM ^g^	CB ^h^	FOX ^i^
PA14	0.25 (0.29)	1 (0.00)	0.125 (0.00)	0.5 (0.00)	2 (0.00)	2 (0.00)	32 (0.00)	256 (0.00)
Δ*glnK*	0.0625 (0.02) *	0.5 (0.00) *	0.0625 (0.00) *	0.5 (0.00)	2 (0.00)	2 (0.00)	32 (0.00)	256 (0.00)
Δ*glnK*/att7::*glnK*	0.25 (0.29)	1 (0.00)	0.125 (0.00)	0.5 (0.00)	2 (0.00)	2 (0.00)	32 (0.00)	256 (0.00)

^a^, The MICs are shown as means of three biological repeats, and values in brackets are standard deviations of the log_2_-transformed MICs. *, *p* < 0.05 compared to wild type PA14 or the complemented strain by one-way ANOVA. ^b^, MEM: meropenem; ^c^, IMP, imipenem; ^d^, DRPM: doripenem; ^e^, FEP: cefepime; ^f^, CAZ: ceftazidime; ^g^, ATM: aztreonam; ^h^, CB: carbenicillin; ^i^, FOX: cefoxitin.

**Table 3:** The role of *oprD* in modulating carbapenem resistance in the Δ*glnK* mutant.

Antibiotics	MIC ^a^ (μg/mL)			
	PA14	Δ*glnK*	Δ*oprD*	Δ*glnK*Δ*oprD*
MEM	0.25 (0.00) *	0.0625 (0.02)	16 (0.00) *	16 (0.00) *
IMP	1 (0.00) *	0.5 (0.00)	8 (0.05) *	8 (0.05) *
DRPM	0.125 (0.00) *	0.0625 (0.00)	4 (0.00) *	4 (0.00) *

^a^, The MICs are shown as means of three biological repeats, and values in brackets are standard deviations of the log_2_-transformed MICs. *, *p* < 0.05 compared to the Δ*glnK* mutant by one-way ANOVA.

**Table 4:** NtrB/NtrC regulates carbapenem susceptibility in the Δ*glnK* mutant.

Antibiotics	MIC (μg/mL)					
	PA14	Δ*glnK*	Δ*ntrB*	Δ*glnK*Δ*ntrB*	Δ*ntrC*	Δ*glnK*Δ*ntrC*
MEM	0.25 (0.00) *	0.0625 (0.02)	0.25 (0.00) *	0.25 (0.00) *	0.25 (0.00) *	0.25 (0.00) *
IMP	1 (0.00) *	0.5 (0.00)	2 (0.29) *	2 (0.29) *	1 (0.00) *	1 (0.00) *
DRPM	0.125 (0.00) *	0.0625 (0.00)	0.125 (0.00) *	0.125 (0.00) *	0.125 (0.00) *	0.125 (0.00) *

The MICs are shown as means of three biological repeats, and values in brackets are standard deviations of the log_2_-transformed MICs. *, *p* < 0.05 compared to the Δ*glnK* mutant by one-way ANOVA.

## Data Availability

Raw RNA-seq datahave been deposited in the National Center for Biotechnology Information (NCBI) Sequence Read Archive (SRA) (https://www.ncbi.nlm.nih.gov/bioproject, accessed on 21 October 2025) under BioProject number: PRJNA1347373.

## Supplementary Materials

The following supporting information can be downloaded at https://www.mdpi.com/article/10.3390/pathogens15030289/s1, Figure S1: mRNA levels of *mexE*, *mexC* and *mexY* in the wild-type PA14 and Δ*glnK*. Gene expression levels were normalized to *rpsL* mRNA levels. Data are shown as mean ± SD from triplicate biological replicates. ns, not significant by one-way ANOVA; Table S1: Differential gene expression of selected gene categories in Δ*glnK* vs. wild-type PA14; Table S2: The role of OprD in the carbapenem resistance of the *ntrB* and *glnK* mutants. Original EMSA gel images (Figure 3B,C).

## Abbreviations

The following abbreviations are used in this manuscript:

MIC	minimum inhibitory concentration
MEM	meropenem
IMP	imipenem
DRPM	doripenem
FEP	cefepime
CAZ	ceftazidime
ATM	aztreonam
CAR	carbenicillin
FOX	cefoxitin
CCCP	carbonyl cyanide m-chlorophenylhydrazone
