# Supplementary material for: Glnk Mediates Carbapenem Resistance Through the NtrB/NtrC-OprD Regulatory Pathway in Pseudomonas aeruginosa
Source: Pathogens. 2026 Mar 6;15(3):289. doi: 10.3390/pathogens15030289 (PMC13029640; doi:10.3390/pathogens15030289)
Supplement: Supplementary file 1 [file pathogens-15-00289-s001.zip › pathogens-4171411-supplementary.pdf]

Supplementary Materials

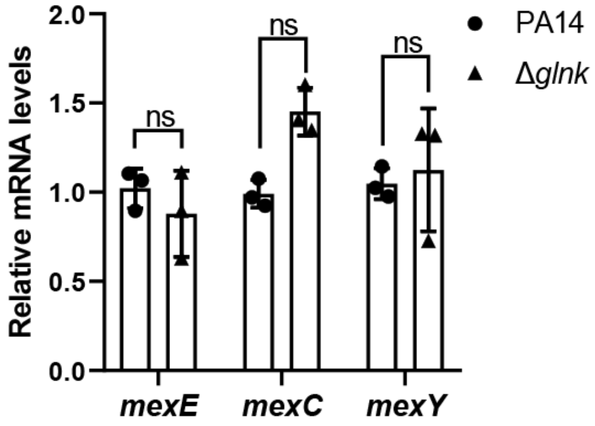

Figure S1. mRNA levels of *mexE*, *mexC* and *mexY* in the wild-type PA14 and Δ*glnK*. Gene expression levels were normalized to *rpsL* mRNA levels. Data are shown as mean ± SD from triplicate biological replicates. ns, not significant by one-way ANOVA.

Table S1. Differential gene expression of selected gene categories in Δ*glnK* vs. wild-type PA14.

Gene ID	Gene Name	Description	log_2FC [a]
		**A.Metabolic genes involved in nitrogen metabolism**	
PA14_60710	*gdhA*	glutamate dehydrogenase	-2.71
PA14_67600	*glnA*	glutamine synthetase	1.82
PA14_49250	*napA*	nitrate reductase catalytic subunit	-1.51
PA14_49210	*napE*	periplasmic nitrate reductase NapE	-0.23
PA14_49230	*napD*	NapD protein of periplasmic nitrate reductase	-1.48
PA14_49250	*napA*	nitrate reductase catalytic subunit	-1.51
PA14_68350	*arcC*	carbamate kinase	-0.40
PA14_49270	*napC*	cytochrome c-type protein NapC	-1.72
PA14_49220	*napF*	ferredoxin protein NapF	-0.18
PA14_03860	-	glutamine synthetase family protein	1.57
PA14_38140	-	glutamine synthetase family protein	1.91
PA14_64350	*ureA*	urease subunit gamma	2.79
PA14_64390	*ureC*	urease subunit alpha	3.08
PA14_64335	*ureD*	urease accessory protein	3.15
PA14_64370	*ureB*	urease subunit beta	3.41
PA14_64660	*ureF*	urease accessory protein UreF	4.94
PA14_64670	*ureG*	urease accessory protein	4.66
PA14_64650	*ureE*	urease accessory protein	4.29
PA14_69795	*amtB*	ammonium transporter	3.29
PA14_44240	-	glutamine synthetase family protein	1.73
PA14_72850	-	glutamine synthetase family protein	1.51

PA14_41540	*nirD*	nitrite reductase small subunit NirD	7.31
**B. RND effux pump and outer membrane porin genes involved in multidrug transport**			
PA14_05530	*mexA*	multidrug efflux RND transporter periplasmic adaptor subunit MexA	0.27
PA14_05540	*mexB*	multidrug efflux RND transporter permease subunit MexB	0.49
PA14_05550	*oprM*	multidrug efflux RND transporter outer membrane channel subunit OprM	0.39
PA14_60850	*mexC*	multidrug efflux RND transporter periplasmic adaptor subunit MexC	0.18
PA14_60830	*mexD*	multidrug efflux RND transporter permease subunit MexD	0.4
PA14_60820	*oprJ*	multidrug efflux transporter outer membrane subunit OprJ	0.27
PA14_32400	*mexE*	multidrug efflux RND transporter periplasmic adaptor subunit MexE	0.26
PA14_32390	*mexF*	multidrug efflux RND transporter permease subunit MexF	0.82
PA14_32380	*oprN*	multidrug efflux RND transporter outer membrane subunit OprN	0.65
PA14_38395	*mexX*	multidrug efflux RND transporter periplasmic adaptor subunit MexX	0.39
PA14_38410	*mexY*	multidrug efflux RND transporter permease subunit MexY	1.12
PA14_51880	*oprD*	outer membrane porin OprD	1.40

[a], FC, fold change.

Table S2. The role of OprD in the carbapenem resistance of the *ntrB* and *glnK* mutants.

**Antibiotics**	**MIC (μg/mL)**					
	**PA14**	**Δ*glnK***	**Δ*ntrB***	**Δ*ntrC***	**Δ*oprD***	**Δ*glnK*Δ*ntrB*Δ*oprD***
MEM	0.25	0.0625	0.25	0.25	16	16
IMP	1	0.5	2	1	8	16
DRPM	0.125	0.0625	0.125	0.125	4	4
