# Supplementary figures and images for: Glnk Mediates Carbapenem Resistance Through the NtrB/NtrC-OprD Regulatory Pathway in Pseudomonas aeruginosa
Source: Pathogens. 2026 Mar 6;15(3):289. doi: 10.3390/pathogens15030289 (PMC13029640; doi:10.3390/pathogens15030289)

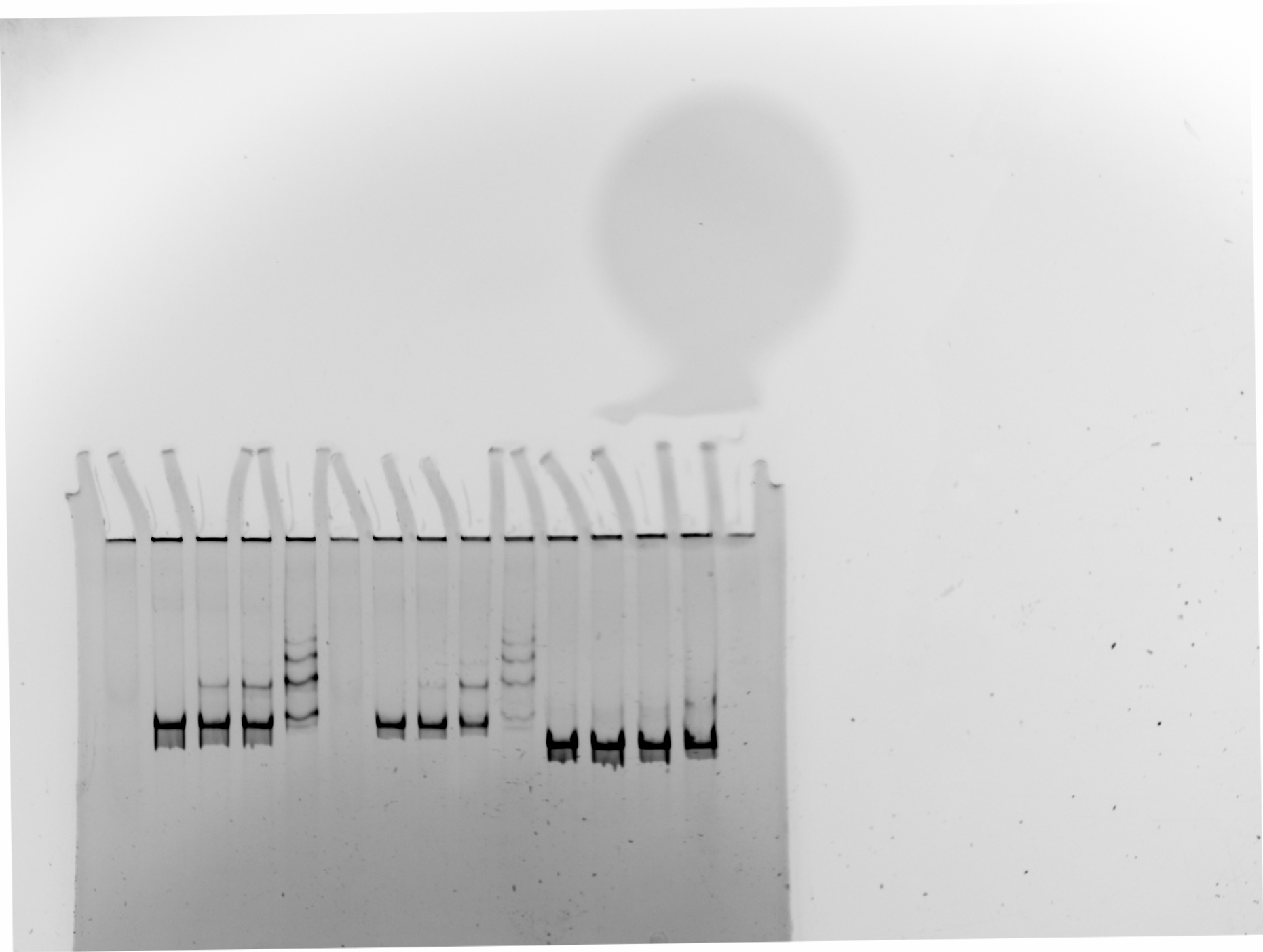

Supplement: Supplementary file 1 [file pathogens-15-00289-s001.zip › Figure 3B.tif]

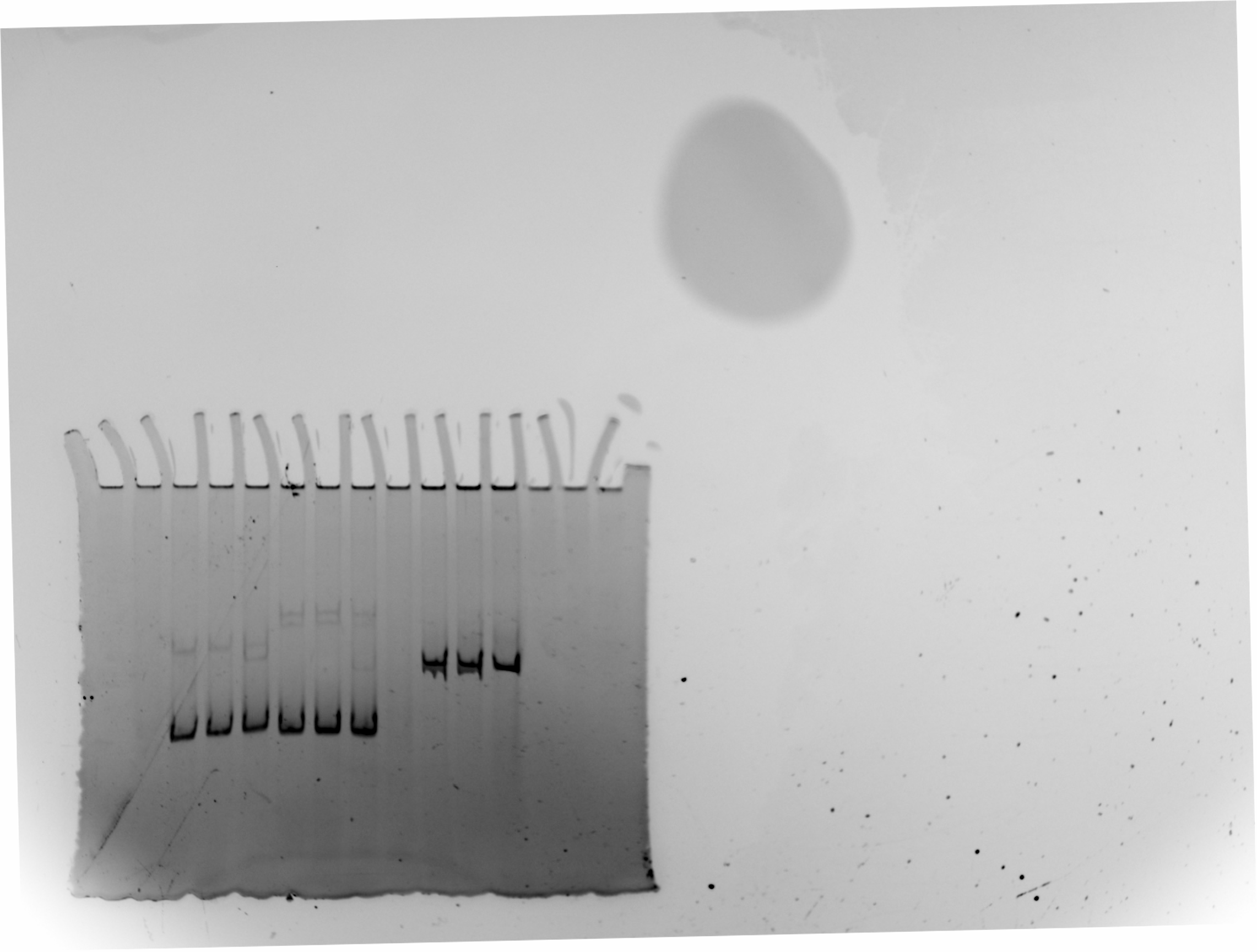

Supplement: Supplementary file 1 [file pathogens-15-00289-s001.zip › Figure 3C.tif]

## Slide 1
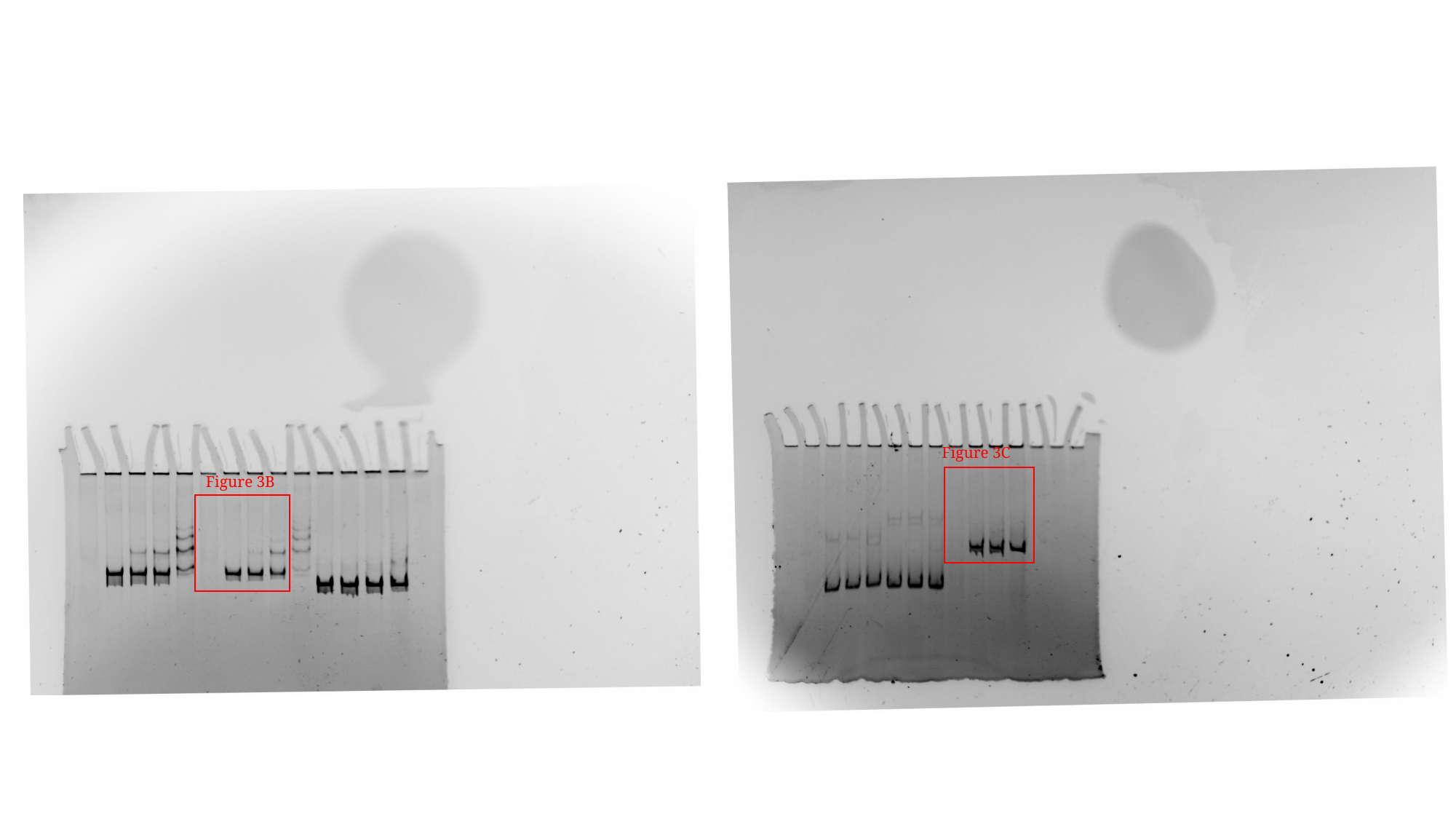

Figure 3C
Figure 3B

Supplement: Supplementary file 1 [file pathogens-15-00289-s001.zip › Original figures.pptx]
